# One-layer versus two-layer duct-to-mucosa pancreaticojejunostomy after pancreaticoduodenectomy: study protocol for a randomized controlled trial

**DOI:** 10.1186/s13063-016-1517-8

**Published:** 2016-08-17

**Authors:** Shu-bo Pan, Wei Geng, Da-chen Zhou, Jiang-ming Chen, Hong-chuan Zhao, Fu-bao Liu, Sheng-xue Xie, Hui Hou, Yi-jun Zhao, Kun Xie, Guo-bin Wang, Xiao-ping Geng

**Affiliations:** 1 Department of Surgery, The Second Affiliated Hospital of Anhui Medical University, Furong Road 678#, Shushan District, Hefei, Anhui 230022 China; 2 Department of Liver Surgery, Ren Ji Hospital, School of Medicine Shanghai Jiao Tong University, No. 1630 Dongfang Road, Shanghai, 200127 China; 3 Department of Surgery, The First Affiliated Hospital of Anhui Medical University, Jixi Road 218#, Shushan District, Hefei, Anhui 230022 China

**Keywords:** Postoperative pancreatic fistula, Pancreaticojejunostomy, Duct-to-mucosa, Pancreaticoduodenectomy

## Abstract

**Background:**

Although various pancreaticojejunal duct-to-mucosa anastomosis methods have been developed to reduce the postoperative risks of pancreaticoduodenectomy, pancreatic fistula remains the most serious complication with a high incident rate. The aim of this study is to compare the safety and effectiveness of one-layer and two-layer duct-to-mucosa pancreaticojejunostomy in patients undergoing pancreaticoduodenectomy.

**Methods/design:**

In this study, adult patients who sign consent forms will be recruited and scheduled for elective pancreaticoduodenectomy. One hundred and fourteen patients will be included and randomized before pancreaticojejunal reconstruction and after resection of the lesion from the pancreatic or periampullary region. The primary efficacy endpoint is the incident rate of postoperative pancreatic fistula. Statistical analysis will be based on the intention-to-treat population. Patients will be followed up for 3 months by monitoring for complications and other adverse events.

**Discussion:**

This prospective, single-center, randomized, single-blinded, two-group parallel trial is designed to compare one-layer with two-layer duct-to-mucosa anastomosis for pancreaticojejunal anastomosis during elective pancreaticoduodenectomy.

**Trial registration:**

Clinical Trials.gov: NCT02511951. Registered on 29 July 2015.

**Electronic supplementary material:**

The online version of this article (doi:10.1186/s13063-016-1517-8) contains supplementary material, which is available to authorized users.

## Background

To date, pancreaticoduodenectomy (PD) has been regarded as the only potentially curative treatment for pancreatic head and periampullary tumors, including tumors in the ampullary region, distal biliary duct, and periampullary duodenum [1]. A retrospective study in which 1000 cases were recruited over the past three decades showed that PD has become an effective treatment to reduce hospital mortality [2]. Mortality has been reduced to less than 5 %, but the morbidity remains at 30–50 % [2, 3]. Postoperative pancreatic fistula (POPF) is one of the most frequent and ominous complications after PD, and its occurrence reportedly ranges from 2–40 % [4, 5]. Severe POPF prolongs hospital stay and requires the use of specific treatments, such as the use of antibiotics, nutritional support, endoscopy, interventional radiology, and/or reoperation, etc. [6]. POPF risk is increased by many factors including pancreatic texture, main pancreatic duct diameter, and pancreaticojejunal (PJ) anastomotic technique [7–9]. Among these factors, only anastomotic technique can be improved. According to the International Study Group of Pancreatic Surgery (ISGPS) definition, POPF exists if the drainage of any measurable volume of fluid containing amylase exceeds three times the normal serum value on or after postoperative day (POD) 3 [10].

Several anastomotic surgical techniques have been developed to reduce the incidence of pancreatic fistula in recent decades, including the duct-to-mucosa method, pancreaticogastrostomy, Peng’s binding method, and the “end-to-end” or “end-to-side” invaginated method. Among these techniques, the conventional duct-to-mucosa method remains the most popular anastomosis due to its advantages. The size of the pancreatic remnant is not limited; moreover, the jejunal lumen and pancreatic remnant lead to easier anastomosis [11–14].

Compared with two-layer duct-to-mucosa anastomosis, the novel one-layer duct-to-mucosa PJ anastomosis method has been reported to be efficient at reducing POPF occurrence [15, 16]. However, the two cited retrospective studies might lead to selection bias. Because this evidence is insufficient, we will conduct a randomized controlled trial to verify the superiority of one-layer duct-to-mucosa PJ anastomosis after PD over the two-layer technique.

## Methods/design

### Study aim

The aim of this study is to compare the effect of two duct-to-mucosa PJ anastomotic methods for PD by assessing factors that are related to mortality or morbidity, including postoperative pancreatic fistula rate, biliary leakage, postpancreatectomy hemorrhage, and anastomosis time.

### Patient involvement

**Fig. 1:**
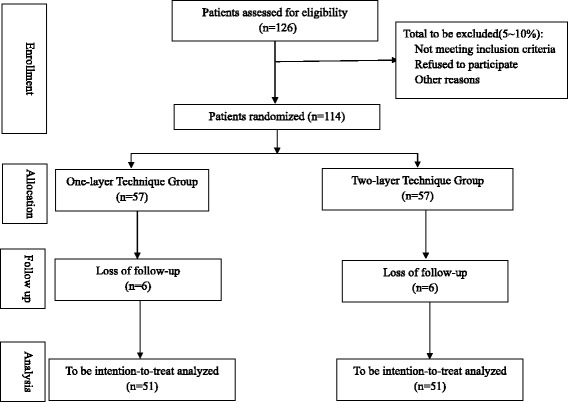
Flow chart according to CONSORT

Sample size calculation is based on the primary endpoint: POPF rate. According to published data, an assumed absolute risk of 22 % difference in POPF occurrence is the appropriate basis for the calculation assuming 4.5 % POPF in the one-layer technique group and 26.7 % in the two-layer technique group [15, 16]. This calculation yields a total of 51 patients in each group, which assures a power of 90 % at a two-sided level of significance of 5 % (NCSS and PASS 11 (NCSS Statistical Software, Kaysville, UT, USA)). Assuming an expected withdrawal rate of 10 % during the trial, 12 additional patients will be included and randomized; therefore, the total sample size required is *n* = 114 patients (Fig. 1) (Additional files 1 and 2).

### Eligibility criteria

#### Inclusion criteria

Patients who meet the following criteria will be included in the study:

1. Age 18–80 years
2. Elective pancreaticoduodenectomy
3. Provision of informed consent.

#### Exclusion criteria

Patients who meet any of the following criteria will be excluded from the study:

1. Patients with any severe cardiopulmonary disease: American Society of Anesthesiologists (ASA) classification or ejection fraction below 30 % that might prolong the postoperative hospital stay
2. Previous pancreatic operation
3. Immunodeficiency, such as that observed under HIV
4. Emergency operation
5. Pregnancy.

#### Withdrawal

Patients can withdraw from the trial at their own request or at the request of their legal representative at any time. Patients may be removed if, in the investigator’s opinion, continuation of the trail could be detrimental to the patient’s well-being or if a PD is not performed due to technical unresectability, metastatic disease, or other reasons. Every withdrawal will be recorded in the clinical report forms (CRFs) and in the patient’s medical case records. All examinations scheduled for the final trial day will be performed on all patients and documented. All data will be analyzed according to the intention-to-treat (ITT) principle [17].

#### Ethics, study registration, and consent

The final protocol has been approved by the Ethics Committee of the Second Affiliated Hospital of Anhui Medical University (approval number: KY201502). The trial protocol has also been registered in the protocol registration system at ClinicalTrials.gov (identifier: NCT02511951). All patients will be scheduled only after comprehensive information concerning the nature, scope, and possible consequences of the clinical trial has been provided to them in an understandable way by the investigator. Written informed consent for the study will be obtained from each patient before the operation. The study procedure, benefits, risks, and data management will be clarified in detail during the preoperative conversation.

## Trial interventions

### One-layer duct-to-mucosa PJ anastomosis

**Fig. 2:**
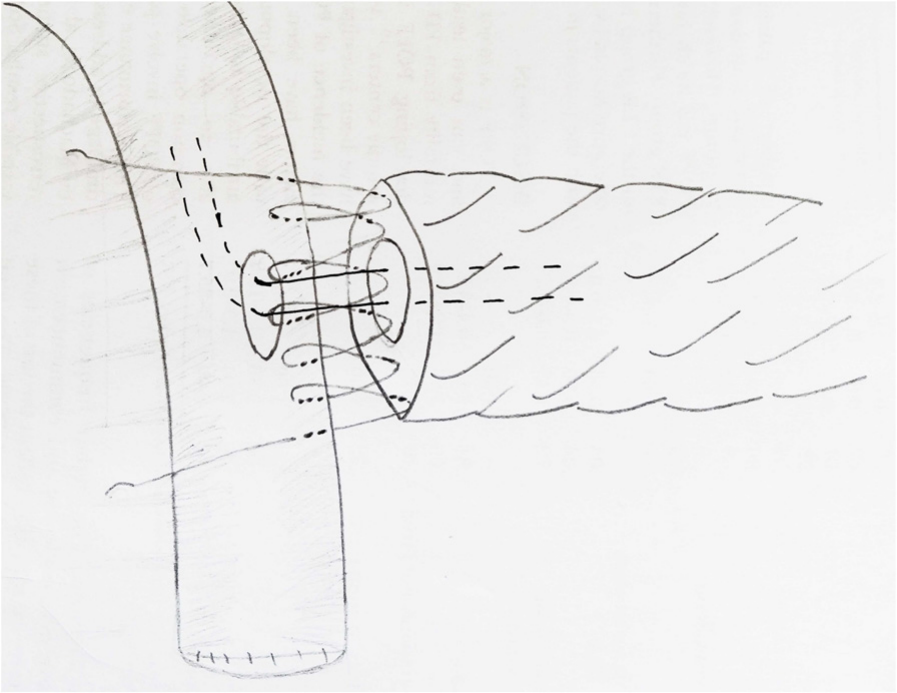
The procedure of end-to-side one-layer duct-to-mucosa PJ anastomosis: the posterior suturing layer

**Fig. 3:**
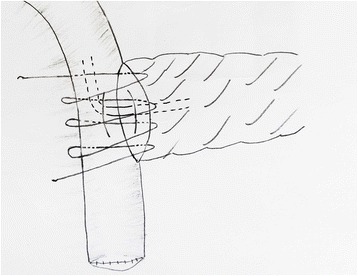
The anterior suturing layer with duct-to-mucosa PJ anastomosis

For the one-layer technique group, to create the posterior suturing layers, double needles with a 4–0 Prolene line (Ethicon, Shanghai, China) will be used; one side of the needles will be inserted from the posterior inner side of the pancreatic duct and out through the dorsal parenchyma of the pancreatic stump to the posterior surface of the pancreas at a point approximately 0.5 cm from the cut edge. The other side of the needles will be started from the inner side of the jejunum lumen, then pushed through the subserosa and seromuscular region, and out from the posterior surface of the bowel (Fig. 2). The anterior suturing layer will be treated in the same manner. An internal pancreatic duct stent will be inserted into the duct of the pancreatic remnant according to its size. Drain tubes will be placed anterior and posterior to the anastomosis (one each) when the anastomosis has been completed (Fig. 3).

### Two-layer duct-to-mucosa PJ anastomosis

**Fig. 4:**
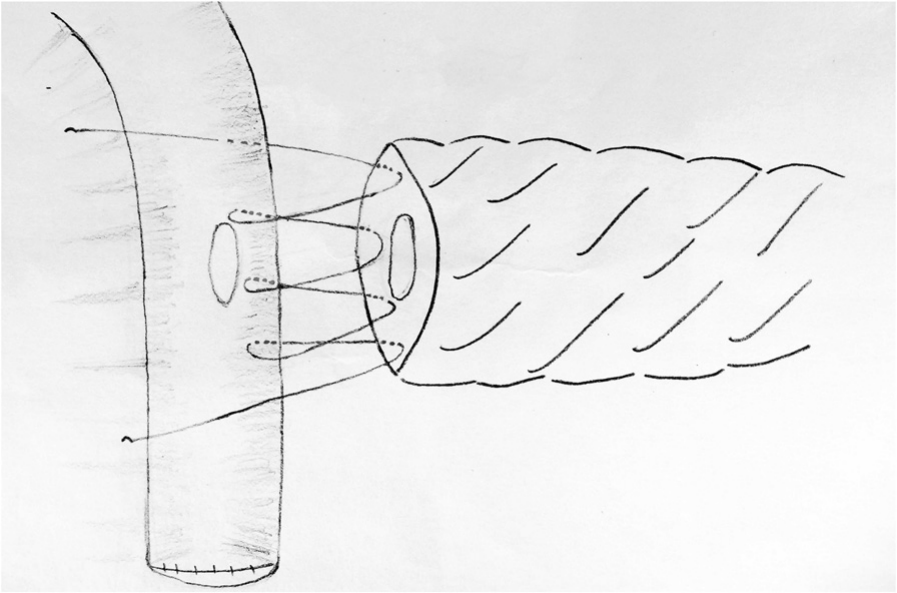
The procedure of end-to-side two-layer duct-to-mucosa PJ anastomosis: the seromuscular layer

**Fig. 5:**
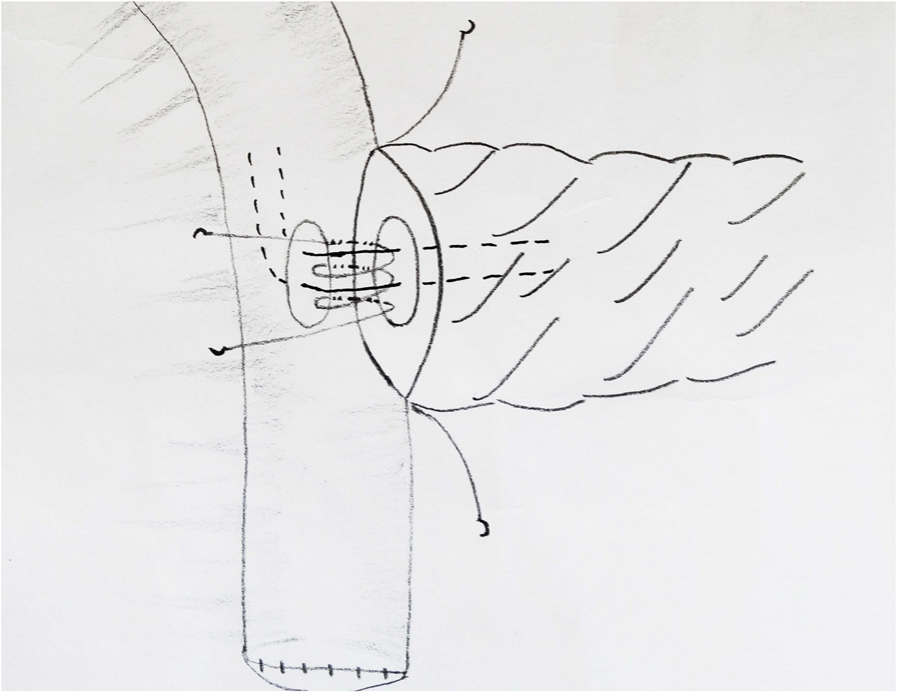
The procedure of two-layer duct-to-mucosa PJ anastomosis: duct-to-mucosa layer

For the two-layer continuous suture method, the same double needle and 4–0 Prolene line will be used. First, the region approximately 1.0 cm from the cutting edge of the pancreatic remnant will be freed; then, the posterior surface of the pancreatic remnant will be sutured to the seromuscular layer of the jejunum using the continuous suturing method (Fig. 4). The jejunum will be brought closer to the stump of the pancreas, and a hole of similar diameter to the main pancreatic duct will be made on the jejunum near the entrance of the main pancreatic duct. The posterior wall of the jejunum near the hole will be sutured to the posterior wall of the pancreatic duct using the continuous suturing method with Prolene line, and a suitable internal pancreatic duct stent will then be placed approximately 4–6 cm into the main duct (Fig. 5). The interior side of the jejunum and pancreas will be sutured using the same method. Then, the anterior surface of the pancreatic remnant and the seromuscular layer of the jejunum will be tightly sutured using the continuous method. Drain tubes will be managed as described above.

### Primary and secondary endpoints

#### Primary endpoint

The primary efficacy endpoint of the trial will be the POPF occurrence rate. POPF is defined by the International Study Group of Pancreatic Fistula (ISGPF) as any measurable volume of drain fluid that contains three times higher amylase content than the normal upper serum value, on or after POD 3. Amylase will be assessed on PODs 1, 3, 5, and 7. Three grades of POPF are determined according to clinical severity: A, B, and C [10].

#### Secondary endpoints

Secondary outcomes will be postoperative hospital stay, anastomosis time, postpancreatectomy hemorrhage, biliary leak, delayed gastric emptying, wound infection, intra-abdominal fluid collection or abscess, relaparotomy, and mortality. Existent ISGPS definitions will be used to ensure the comparability and generalizability of the results (Table 1). Postoperative complications will be graded based on severity according to the Clavien-Dindo definition [18] (Table 2).

**Table 1:** Definition of secondary endpoints

Secondary endpoint	Definition and assessment of outcomes
Anastomosis time	Time from beginning to end of PJ anastomosis
Mortality	Death due to any cause until 90 days after the operation and the reason
Morbidity	Postoperative complications will be recorded until 90 days after operation. The severity of complications will be graded according to the Clavien-Dindo classification [18]
Postoperative hospital stay	Time from day of operation until discharge (days)
Postpancreatectomy hemorrhage	Evidence of blood loss from drains and/or nasogastric tube, based on ultrasonography, as defined by ISGPS [19]
Biliary leak	Bilirubin concentration in the drain fluid at least three times the serum bilirubin concentration as defined by International Study Group of Liver Surgery [20]
Delayed gastric emptying	Failure to resume solid diet with prolonged need for nasogastric tube as defined by ISGPS [21]
Intra-abdominal fluid collection	Collection of fluid measuring ≥3 cm associated with clinical or laboratory abnormalities
Wound infection	Surgical site infection associated with laparotomy that develops during the initial hospital stay
Pneumonia	Presence of a new infiltrate on chest X-ray, as well as following: body temperature >38 °C, abnormal elevation of white blood cells, or positive sputum, and requiring antibiotic treatment
Abdominal rupture	Dehiscence of abnormal closure with need for resuture of the laparotomy during the initial hospital stay

**Table 2:** Complication grades according to the Clavien-Dindo classication scheme^a^

Grade	Definition
I	Any deviation from the normal postoperative course without the need for pharmacological treatment or surgical, endoscopic, and radiological intervention
II	Requiring pharmacological treatment with drugs other than those allowed for grade I complications
III	Requiring surgical, endoscopic, or radiological intervention
IIIa	Intervention not under general anesthesia
IIIb	Intervention under general anesthesia
IV	Life-threatening complication. Requiring intensive care unit management
IVa	Single organ dysfunction
IVb	Multi-organ dysfunction
V	Death of patient

^a^The Clavien-Dindo classification system is reported in detail elsewhere [18]

### Type of trial

This is a prospective, randomized, interventional, and patient-blinded, single-center trial comparing two parallel groups.

#### Randomization

To achieve intervention groups with comparable known and unknown risk factors, randomization will be performed. The randomization number will be generated using computer-generated random numbers with an allocation ratio of 1:1 (an equal probability of assignment to either group). All patients will be randomized using consecutively numbered opaque envelopes that will have been sealed by the investigators. The envelopes will be opened before PJ reconstruction and after resection of the lesion from the pancreatic or periampullary region.

#### Blinding

Patients and outcome observers will be blinded with respect to the trial intervention. Blinding of the surgeons and people involved in the operation is not feasible due to the nature of the interventions.

### Data management and quality assurance

An independent study doctor (SBP), who will not be involved in the treatment and monitoring of the patients within the operating room, will enter all required data in the prepared CRF. This CRF will be completed as soon as possible, preferably on the day of the patient’s treatment and visit (Table 3). Reasonable explanations should be given for all missing data. Complete CRF pages will be checked by the principal investigator and the responsible monitor with respect to completeness and plausibility.

**Table 3:** Flow chart of the trial

	Screening					
	Visit 1	Visit 2	Visit 3	Visit 3	Visit 3	Visit 4
	Before surgery	Day of surgery	(POD^b^ 1)	(POD 3)	(POD 7)	(POD 90)
Selection criteria						
Informed consent	×					
Past medical history						
Personal data^a^	×					
Physical examination	×					
Laboratory tests	×		×	×	×	×
Trial intervention		×				
Intraoperative outcomes		×				
Postoperative outcomes			×	×	×	×

^a^Height (cm), weight (kg), gender, immunosuppressant medication, antibiotics, chemotherapy

^b^Postoperative day

### Statistical analysis

The two-sided null hypothesis for the primary endpoint measurement states that both study interventions will lead to a similar POPF occurrence rate; the alternative hypothesis is that one intervention will perform better than the other. The null hypothesis will be tested by analyzing the covariance while adjusting for pancreatic texture (soft or hard) and main pancreatic duct diameter (<3 mm or ≥3 mm). A binary logistic regression will be applied to compare the POPF occurrence rates between the groups after adjusting for other factors. Background characteristics and surgical outcome measures will be compared using chi-squared or Fisher’s exact tests for categorical data and two-tailed *t* tests or nonparametric Mann–Whitney *U* tests for continuous variables. Categorical data will be presented as frequencies and group percentages, and continuous variables will be expressed as the means and standard deviations. The homogeneity of two groups will be described by comparison of the demographic data and baseline values. All analyses will be performed on an ITT basis [17]. For the ITT analysis, the data will be processed for all trial patients in their randomized groups. A *P* value < 0.05 will be considered statistically significant. All statistical calculations will be performed using SPSS10.0 (SPSS, Chicago, IL, USA).

## Discussion

Currently, pancreaticoduodenectomy is a routine operation, and postoperative mortality is less than 5 % [2, 3]. POPF is among the most frequently encountered complications that contribute to a high postoperative mortality. Debate regarding the preferred surgical technique for PJ anastomosis has continued for decades. Many retrospective studies have suggested that the POPF occurrence rate is reduced by using a one-layer rather than a two-layer duct-to-mucosa PJ anastomosis [15, 16]. However, more reliable evidence should be accumulated to address the advantages and disadvantages of both techniques. In this way, the most beneficial technique can be selected for individual patients. Therefore, the factors affecting the success of one-layer versus two-layer duct-to-mucosa PJ anastomosis should be evaluated in this randomized controlled trial to minimize the POPF rates associated with PD.

## Trial status

The trail is currently recruiting patients. All patients should be recruited by December 2018.

## Abbreviations

CRF, clinical report form; ISGLS, International Study Group of Liver Surgery; ISGPF, International Study Group of Pancreatic Fistula; ISGPS, International Study Group of Pancreatic Surgery; POPF, postoperative pancreatic fistula; WBC, white blood cell

## Additional files

Additional file 1: SPIRIT checklist. (DOC 119 kb) Additional file 2: SPIRIT Figure. (DOC 13 kb)

## References

[1] Hartwig W, Werner J, Jäger D, Debus J, Büchler MW (2013). Improvement of surgical results for pancreatic cancer. Lancet Oncol.

[2] Cameron JL, Riall TS, Coleman J, Belcher KA (2006). One thousand consecutive pancreaticoduodenectomies. Ann Surg.

[3] Čečka F, Jon B, Šubrt Z, Ferko A (2013). Clinical and economic consequences of pancreatic fistula after elective pancreatic resection. Hepatobiliary Pancreat Dis Int.

[4] De Carlis L, Ferla F, Di Sandro S, Giacomoni A, De Carlis R, Sguinzi R (2014). Pancreatico-duodenectomy and postoperative pancreatic fistula: risk factors and technical considerations in a specialized HPB center. Updates Surg.

[5] Zhang T, Xu J, Wang T, Liao Q, Dai M, Zhao Y (2013). Enucleation of pancreatic lesions: indications, outcomes, and risk factors for clinical pancreatic fistula. J Gastrointest Surg.

[6] Ramacciato G, Mercantini P, Petrucciani N, Nigri GR, Kazemi A, Muroni M (2011). Risk factors of pancreatic fistula after pancreaticoduodenectomy: a collective review. Am Surg.

[7] Chen Y, Ke N, Tan C, Zhang H, Wang X, Mai G (2015). Continuous versus interrupted suture techniques of pancreaticojejunostomy after pancreaticoduodenectomy. J Surg Res.

[8] Xu J, Zhang B, Shi S, Qin Y, Ji S, Xu W (2015). Papillary-like main pancreatic duct invaginated pancreaticojejunostomy versus duct-to-mucosa pancreaticojejunostomy after pancreaticoduodenectomy: a prospective randomized trial. Surgery.

[9] Nojiri T, Misawa T, Saitoh R, Shiba H, Usuba T, Uwagawa T (2011). Technical and mechanical risk factors for postoperative pancreatic fistula in pancreaticojejunostomy. Hepatogastroenterology.

[10] Bassi C, Dervenis C, Butturini G, Fingerhut A, Yeo C, Izbicki J (2005). Postoperative pancreatic fistula: an international study group (ISGPF) definition. Surgery.

[11] Osman MM, Abd E, Maksoud W (2014). Evaluation of a new modification of pancreaticogastrostomy after pancreaticoduodenectomy: anastomosis of the pancreatic duct to the gastric mucosa with invagination of the pancreatic remnant end into the posterior gastric wall for patients with cancer head of pancreas and periampullary carcinoma in terms of postoperative pancreatic fistula formation. Int J Surg Oncol..

[12] Peng SY, Wang JW, Li JT, Mou YP, Liu YB, Cai XJ (2004). Binding pancreaticojejunostomy — a safe and reliable anastomosis procedure. HPB (Oxford).

[13] Maemura K, Mataki Y, Kurahara H, Mori S, Higo N, Sakoda M (2015). Pancreaticogastrostomy after pancreaticoduodenectomy using twin square wrapping with duct-to-mucosa anastomosis. Eur Surg Res.

[14] El Nakeeb A, El Hemaly M, Askr W, Abd Ellatif M, Hamed H, Elghawalby A (2015). Comparative study between duct to mucosa and invagination pancreaticojejunostomy after pancreaticoduodenectomy: a prospective randomized study. Int J Surg.

[15] Wei J, Liu X, Wu J, Xu W, Zhou J, Lu Z (2015). Modified one-layer duct-to-mucosa pancreaticojejunostomy reduces pancreatic fistula after pancreaticoduodenectomy. Int Surg.

[16] Zhang L, Li Z, Wu X, Li Y, Zeng Z (2015). Sealing pancreaticojejunostomy in combination with duct parenchyma to mucosa seromuscular one-layer anastomosis: a novel technique to prevent pancreatic fistula after pancreaticoduodenectomy. J Am Coll Surg.

[17] Montedori A, Bonacini MI, Casazza G, Luchetta ML, Duca P, Cozzolino F, Abraha I (2011). Modified versus standard intention-to-treat reporting: are there differences in methodological quality, sponsorship, and findings in randomized trials? A cross-sectional study. Trials..

[18] Dindo D, Demartines N, Clavien PA (2004). Classification of surgical complications: a new proposal with evaluation in a cohort of 6336 patients and results of a survey. Ann Surg.

[19] Wente MN, Veit JA, Bassi C, Dervenis C, Fingerhut A, Gouma DJ (2007). Postpancreatectomy hemorrhage (PPH): an International Study Group of Pancreatic Surgery (ISGPS) definition. Surgery.

[20] Koch M, Garden OJ, Padbury R, Rahbari NN, Adam R, Capussotti L (2011). Bile leakage after hepatobiliary and pancreatic surgery: a definition and grading of severity by the International Study Group of Liver Surgery. Surgery.

[21] Wente MN, Bassi C, Dervenis C, Fingerhut A, Gouma DJ, Izbicki JR (2007). Delayed gastric emptying (DGE) after pancreatic surgery: a suggested definition by the International Study Group of Pancreatic Surgery (ISGPS). Surgery.

