# Supplementary material for: One-layer versus two-layer duct-to-mucosa pancreaticojejunostomy after pancreaticoduodenectomy: study protocol for a randomized controlled trial
Source: Trials. 2016 Aug 17;17:407. doi: 10.1186/s13063-016-1517-8 (PMC4988010; doi:10.1186/s13063-016-1517-8)
Supplement: Additional file 2: — SPIRIT Figure. (DOC 13 kb) [file 13063_2016_1517_MOESM2_ESM.docx]

| Table flowchart of the trial | | | | | | | |
| --- | --- | --- | --- | --- | --- | --- | --- |
| Screening | | | | | | | |
|  | Visit 1 before surgery | Visit 2 after surgery | Visit 3 (POD1) | Visit 4 (POD3) | Visit 5 (POD5) | Visit 6 (POD7) | Visit 7 (POD90) |
| Selection criteria and informed consent | X |  |  |  |  |  |  |
| Medical history Demographics | X |  |  |  |  |  |  |
| Physical examination | X |  |  |  |  |  |  |
| Laboratory texts | X |  | X | X | X | X | X |
| Trial intervention |  | X |  |  |  |  |  |
| Intraoperative outcomes |  | X |  |  |  |  |  |
| Postoperative outcomes |  |  | X | X | X | X | X |
